# Vision for the Future Project: Screening impact on the prevention and treatment of visual impairments in public school children in São Paulo City, Brazil

**DOI:** 10.6061/clinics/2021/e3062

**Published:** 2021-09-20

**Authors:** Douglas Rodrigues da Costa, Iara Debert, Fernanda Nicolela Susanna, Janaina Guerra Falabreti, Mariza Polati, Remo Susanna

**Affiliations:** Faculdade de Medicina FMUSP, Universidade de Sao Paulo, Sao Paulo, SP, BR.

**Keywords:** Visual Screening, Refractive Error, Ophthalmic Epidemiology, Amblyopia, Social Refraction

## Abstract

**OBJECTIVES:**

Uncorrected refractive errors are the leading cause of visual impairment in children. In this cross-sectional retrospective study, we analyzed a social visual screening program for school children in São Paulo, Brazil, evaluated its impact on the prevention and treatment of children’s visual disabilities, and assessed its epidemiological outcomes to outline suggestions for its improvement.

**METHODS:**

First-grade children from public schools were submitted to prior visual screening by their teachers. Selected children were forwarded to the hospital’s campaigns for a second screening by ophthalmologists and treatment if needed. Data were analyzed for age, sex, visual acuity, biomicroscopy, refractive errors, ocular movement disorders, amblyopia, number of donated spectacles, and number of children forwarded to specialized care.

**RESULTS:**

A total of 1080 children were included with mean age of 6.24±0.45 years. Children with normal ophthalmological exam, 591 (54.7%; 95% confidence interval [CI]: 51.7%-57.7%) were dismissed and considered false-positives. Myopia, hyperopia, and astigmatism components were found in 164 (15.2%; CI: 13.1%-17.4%), 190 (17.6%; CI: 15.3%-20.0%), and 330 (30.5%; CI: 27.8%-33.4%) children, respectively. Amblyopia was diagnosed in 54 (5%; CI: 3.5%-6.4%) children, and 117 (10.8%; CI: 9.8%-12.8%) presented ocular movement disorders. A total of 420 glasses were donated.

**CONCLUSION:**

Epidemiological findings for amblyopia and refractive errors are consistent with those of similar studies. The expressive number of diagnoses performed and number of glasses donated to underprivileged children depict the importance of such projects. New guidelines to improve their cost-effectiveness, such as professional training and community sensitization, are imperative.

## INTRODUCTION

In 1999, the World Health Organization (WHO) initiated the project “Vision 2020 - The Right to Sight” to combat blindness and low vision. The goal was to eliminate preventable blindness by 2020 (1). Despite the efforts worldwide, recent meta-analyses have pointed to an increasing number of avoidable blindness, primarily because of cataracts and uncorrected refractive errors (myopia, hyperopia, and astigmatism) (2,3). Non-developed countries account for the largest share of these cases, as they present more obstacles to reaching quality health units. According to the last “Human Development Report 2019” from the United Nations Development Programme, scholars’ level and income are directly proportional to health access. In Brazil, blindness and low vision are approximately three times more in the poor than in the rich population (4). Considering pre-scholar and scholar populations, approximately 20%-30% of children have an ophthalmologic condition that can lead to blindness or low vision, particularly uncorrected refractive errors.

Vision plays a significant role in a child’s development. Healthy visual maturation in the early years of a child’s life is responsible for creating permanent connections to form neuro-ophthalmological pathways (5,6). Precocious detection and treatment of ophthalmological disturbances that might harm visual maturation are critical to enhancing various abilities such as motor skills, reading, and writing, as well as for self-esteem and quality of life (5,7,8). Furthermore, regions with a higher prevalence of untreated ophthalmic conditions have worse socioeconomic indicators (2,4). In this context, social programs for visual screening in the early years have become a tool for tracing, diagnosing, and treating ophthalmological affections, particularly for children within that socioeconomic portion (9).

The project “Vision for the Future” is a social action that was initiated in 2009 in São Paulo, Brazil. It has been promoted by government educational entities and created to reach underprivileged children who are enrolled in the early phases of elementary school. The main goal of this program is based on visual screening, free medical aid, and donation of corrective spectacles, if necessary. This study aimed to describe the “Vision for the Future” program and to evaluate its impact on the prevention and treatment of children’s visual disabilities, outline suggestions for the program’s improvement, and furnish new epidemiological data to the studied population.

## METHODS

This was a retrospective, cross-sectional, and observational study. Data collected from 2018’s “Vision for the Future” campaigns were analyzed. The protocols of this study were elaborated according to the Declaration of Helsinki and approved by the University’s Ethical Committee on Research. APPROVAL NUMBER (Plataforma Brasil): CAAE: 29047520.9.0000.0068.

### Program description

**Figure 1:**
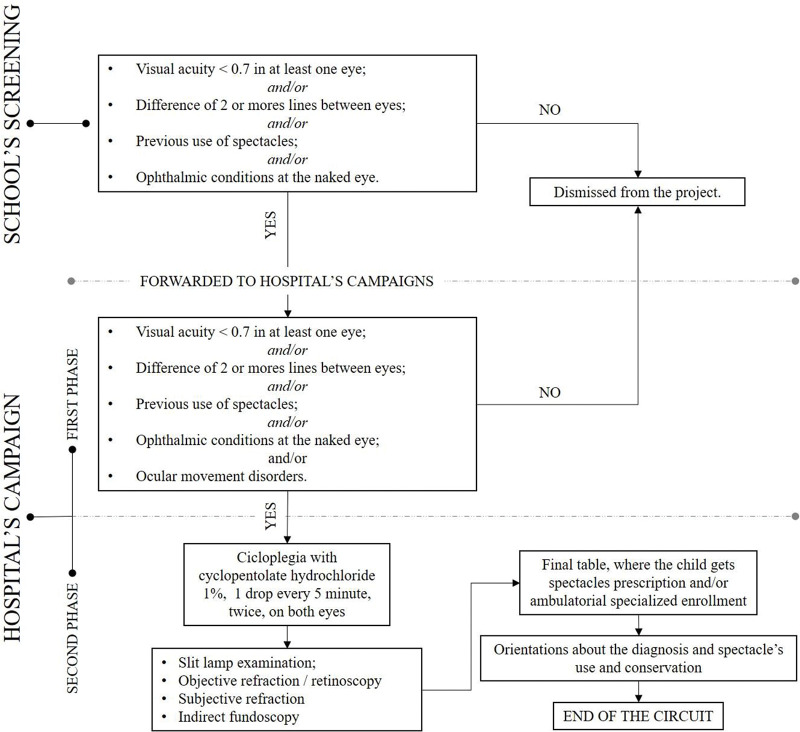
“Vision for the Future” examination circuit in the year 2018.

The program initiated with the public, municipal, or federal schools from any region within São Paulo city. It was divided into two main parts: School’s Screening and Hospital’s Campaigns (Figure 1). In the first part, the children were evaluated by teaching professionals using the Snellen Chart visual acuity (VA) test and naked eye examination. They were referred to the Hospital’s Campaigns if they presented at least one of the following: VA <0.7 (decimal score) and/or a difference equal to or bigger than two lines between eyes and/or visible ophthalmic disturbs at the naked eye (corneal and/or palpebral alterations, strabismus, and others) and/or previous use of spectacles.

The Hospital’s Campaigns took place in a public hospital in São Paulo. Ophthalmologists and ophthalmology residents performed the examinations, and other professionals from the Department of Ophthalmology were responsible for the organization of the examination circuit. Children were accompanied by their guardians who possessed a unique file to be filled by the medical staff. The Hospital’s Campaigns were also divided into two phases.

In the first phase, children underwent a VA test using the Snellen chart, ocular movement tests, and a naked eye examination (ectoscopy). The cutoffs were the same as those in the School’s Screening, as aforementioned. Children who were classified as normal in the first phase were considered false positives since they should not be referred. The second phase comprised a slit-lamp examination, objective refraction (automated and/or retinoscopy), subjective refraction, and a fundoscopy examination, all performed after cycloplegia (cyclopentolate hydrochloride 1%, 1 drop every 5 min, twice). All results were then submitted to a final table, where ophthalmologists decided whether the child needed spectacle prescription and/or referral to a specialized follow-up. All glasses were given free, and they could be withdrawn from the schools.

### Data analysis

Children's files were transferred to an online platform, Research Electronic Data Capture (REDCap), and analyzed using IBM SPSS Statistics 24 (IBM, Endicott, New York, USA) (10,11). All data collected from January to December 2018 were included in the analyses. The investigators that evolved in the project had full access to the data. Patients with missing data were excluded from the study. Confidence intervals (CIs) with a 95% confidence level were calculated using the binomial “exact” method. As patients were pre-selected on school screening, an intrinsic selection bias existed; therefore, statistical analysis for epidemiological extrapolation was not performed.

Data obtained included sex; age; the number of children dismissed on the First Phase of Hospital’s Campaigns (false positives); previous use of spectacles; VA with and without refractive correction; refractive errors (spherical, cylinder, and axis); slit lamp and fundoscopy findings; ocular movement disorders; and the number of glasses donated. Visual impairment is generally classified as a decrease in VA caused by any condition (12). Low vision is characterized as a VA of ≤0.3 and legal blindness as a VA of ≤0.1 (13). To classify ametropia, the following values were considered: hyperopia as a cylinder power >+2.00 diopters (D), myopia if <-0.50 D, and astigmatism if the cylinder power was at least -0.75 D (14). Astigmatism was also classified as: “with the rule” astigmatism when the weakest refractive power was between the axis of 20° and 160°; “against the rule” when between 70° and 110°; and as obliquus if the axis was elsewhere (14). Finally, we calculated the number of amblyopia detected, for which the definition was of two or more lines of difference in VA between the eyes, which could not be explained by other subjacent conditions (15).

## RESULTS

**Figure 2:**
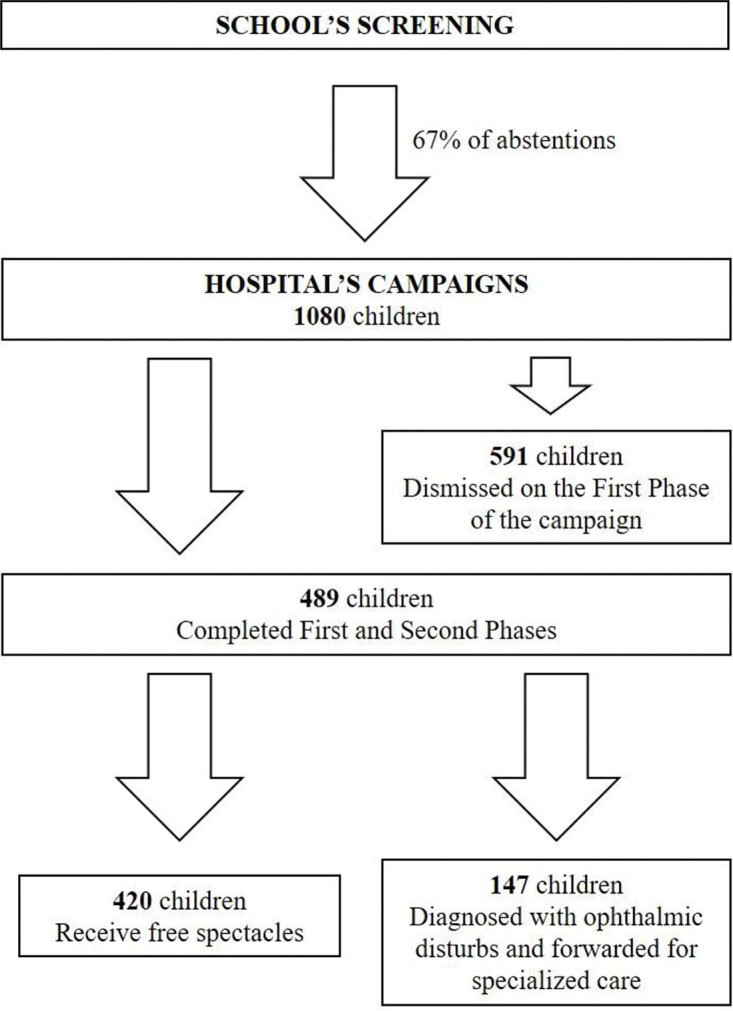
“Vision for the Future” children’s participation flowchart for the year 2018.

Data from 1080 children who went to Hospital’s Campaigns in 2018 were analyzed. All children were born in Brazil and were currently studying in public schools in São Paulo city. There were 534 boys (49.4%) and 546 girls (50.6%). The mean age was 6.24±0.45 years, the youngest aged 6 years, and the oldest aged 9 years. The distribution of VA without refractive correction is shown in Table 1. It was found that 117 (10.8%; CI: 9.0%-12.8%) children had ocular movement disorders, with tropias being the most prevalent (26.4% of esotropias and 25.6% of exotropias, Table 2). A total of 489 children (45.3%; CI: 42.3%-48.3%) were selected in the second phase, and 52 of these already made use of spectacles (Figure 2). Therefore, 591 (54.7%, 95% CI: 51.7%-57.7%) were wrongly selected on School’s Screening and were classified false positives.

In the second phase of the Hospital’s Campaigns, 489 selected children were analyzed. Of these, 164 (33.5%) presented a myopic component, 190 (38.8%) presented a hyperopic component, and 330 (67.4%) had an astigmatism component (79.5% with the rule, 5.8% against the rule, and 14.6% obliquus). When compared with the total 1080 children, the refractive error distributions were 15.2%, 17.6%, and 30.5%, respectively. The total number of children with amblyopia was 54 (5%; CI: 3.1%-6.4%). VA analyses showed that 114 (10.5%) children presented a VA of ≤0.3; hence, they were classified as having low vision. Of the 114 children with low vision, 94 (82.4%; CI: 74.4%-88.9%) achieved normal vision after refractive correction using spectacles. A total of 220 functional and structural alterations were diagnosed, as shown in Table 3. At the end of the circuit, 147 (13.6%; CI: 11.6%-15.8%) children were referred for specialized follow-up, 420 (38.8%; CI: 35.9%-41.8%) glasses were donated, and 342 children could be dismissed with a normal ophthalmological examination after the spectacles were donated.

## DISCUSSION

In this study, we described the social project “Vision for the Future” and analyzed its epidemiological data in 2018. We intend to outline new proposals to enhance this project and others alike and to supplement the current literature, as there is a lack of new epidemiological data for this population, mainly in developing countries. The results of this report corroborate those of previous studies and demonstrate the importance of screening programs for children (12,16).

Meta-analytical data and WHO reports indicate that uncorrected refractive errors are the leading cause of visual impairment and second leading cause of blindness worldwide, affecting approximately 102 million individuals and blinding up to 6 million individuals of all ages (2,17,18). The prevalence of visual impairments and reversible blindness is higher in low-and middle-income countries, and it has an inversely proportional curve with health investments. Uncorrected refractive errors and visual impairments in childhood are even more important as they can lead to amblyopia (15). Since the amblyopic eye will no longer develop its full VA potential, this pathology may lead to irreversible visual impairment or even blindness if not properly treated (5).

In our cohort, we detected amblyopia in 5% (95% CI: 3.5%-6.4%) of the children. The prevalence of this effect is highly dependent on the region, age, and socioeconomic status (19). Meta-analytic studies, including all ages, have shown a pooled prevalence estimate of amblyopia of 1.75% (95% CI: 1.17%-1.88%), ranging from 0.51% to 3.67% (20). Another systematic review had a similar result, with a pooled estimate of 1.44% (95% CI: 1.17%-1.78%) and prevalence of 2.90%, 2.41%, 1.09%, and 0.72% in Europe, North America, Asia, and Africa, respectively (21). These numbers range from 1% to 3% in childhood worldwide (22). The prevalence in our study was higher since our cohort was previously selected by an initial school screening. All patients were forwarded for specialized follow-up and treatment.

Amblyopia treatment should ideally begin between 2 and 8 years of age. During that period, crucial neuronal connections are made to form neuro visual paths that will remain for life (15,23,24). In this social project, children’s ages ranged from 6 to 9 years. Although the detection and treatment are still efficient, younger children should also be screened and included in this and other screening programs (25-27). The major obstacle is the difficulty in examining the preverbal and illiterate. Larger investments would be required to cover the extra population, which is hardly a viable option (28). New instruments for photorefraction, such as smartphone-based applications, are promising for this purpose. These devices exhibit high-performance metrics for the detection of amblyopia risk factors such as ametropias and strabismus (28-31).

Photorefractive devices have been compared with VA testing in infants for amblyopia screening. For younger elementary children and for those with special needs, the sensitivity and specificity of photoscreening are considerably higher than those of patched VA testing (32). An Alaskan study reported two different devices that achieved sensitivity/specificity of 77%/99% and 85%/99%, respectively, while that of VA testing was only 39%/99% (32). In addition, approximately a third of the pre-scholar children could not complete the VA test because of a lack of cooperation. Additionally, a retrospective analysis of the photoscreening of 21367 children showed better results for detecting amblyopia, particularly owing to the possibility of examining younger children (33). Children were treated earlier and presented better visual results during the follow-up. These results corroborate the idea of using photorefractive devices to detect refractive errors and amblyopia risk factors.

Uncorrected refractive error was the most prevalent finding in this study. Within our 1080 children cohort, we found 15.2%, 17.6%, and 30.5% of myopia, hyperopia, and astigmatism components, respectively. A meta-analytic study showed that for children, the globally estimated pooled prevalence of myopia, hyperopia, and astigmatism was 11.7%, 4.6%, and 14.9%, respectively (17). In a Brazilian cohort of patients aged <10 years, the prevalence of myopia, hyperopia, and astigmatism was 3.8%, 86.9%, and 25%, respectively (34). The cutoff values the authors used were a cylinder power of at least -0.50 D and +0.50 D for myopia and hyperopia, respectively, and a cylinder power of -0.50 D for astigmatism. The reason for the remarkably high prevalence of hyperopia can be explained by the lower cutoff adopted by the authors. The prevalence of myopia is surprisingly lower than that reported in our findings and global estimated pools, which is closer to our findings. The prevalence of the astigmatism component was similar to that reported in a Malaysian study (21.3%) (35). Finally, a total of 420 (38.8%; 95% CI: 35.9%-41.8%) spectacles were donated in 2018.

The strategy of donating spectacles is one of the most important factors for compliance in refractive social campaigns, particularly in developing countries (16). Our data suggested that approximately 67% of the children selected for the Hospital’s Campaigns did not show up. All children who were forwarded to the hospital were taken from their schools by pre-paid charter buses by governmental institutions. Therefore, approximately two-thirds of the expenses in that manner were wasted. Additionally, we found a high number of false positives that were screened by teaching professionals. From 1080 children evaluated in the First Phase of the Hospital’s Campaigns, 591 (54.7%, 95% CI: 51.7%-57.7%) were dismissed and classified false positives. Together, these two factors point to an exceptionally low cost-effective screening program. Besides economics, two major problems are highlighted: lack of compliance by parents and children, and teaching professionals’ poor ophthalmic examination training.

School-based screening programs have been demonstrated to be more cost-effective than other primary eye care models (36). Additionally, teaching professionals can be trained and qualified for visual screening (37,38). However, it is costly to train all the teachers. However, simultaneously, it has been shown that more teachers doing screening improve the rates of true positives (39,40). In our cohort, false-positive rates were three to five times higher than those found in an Indian study, which found the rates to be 16.6% and 9.7%, respectively, when adopting the method of screening by only a few selected teachers or by all school teachers, respectively (39). As the literature indicates, the number of children screened per teacher must be lowered. Therefore, new ophthalmic programs for teaching professionals’ capacitation must be implemented. Another option is the adjoint use of smartphone-based photorefractive applications mentioned above, which reduces the time of screening, particularly for less cooperative individuals (28-30).

Another key aspect to improve social programs is the compliance of the population. The expressive amount of children’s absence suggests poor community sensitization. Good community sensitization is critical for the success and sustainability of screening programs. Teachers, parents, care holders, and children must be aware of the reasons and benefits of such programs. Methods to address specific communities must be adopted, such as posters, the use of radio and television, and communication via religious groups and community leaders. The awareness of the goals and targets of the programs will yield results and long-term compliance to the screening program, leading to better community health indices.

The main limitation of this study is the impossibility of statistical extrapolation, as with boot-strap techniques, since our cohort was already pre-selected and school screening was considered a selection bias. However, as discussed above, the epidemiological findings in this study are consistent with those of previous studies and can be considered in our population. In addition, we could not measure the impact of the treatment for children with conditions that required longer follow-ups, such as amblyopia. Nonetheless, the number of donated spectacles and high ratio of children who acquired normal vision after visual treatment states the impact of such programs on visual rehabilitation. Finally, epidemiological data regarding this population in Brazil are scarce and outdated; thus, trustworthy sources of information should be shared.

In conclusion, this study intended to describe the “Vision for the Future” social program, to analyze its epidemiological outcomes, and to outline suggestions for the improvement of this program and others alike. School-based screening programs are essential for eye care settings, especially in developing countries. More studies are needed to improve the cost-effectiveness and efficiency of social and screening programs.

## AUTHOR CONTRIBUTIONS

Costa DR was responsible for the manuscript elaboration and revision, and data collection and analysis. Debert I was responsible for the manuscript elaboration and revision, and orientation. Susanna FN was responsible for the manuscript elaboration and revision. Falabreti JG was responsible for the data collection and analysis. Polati M and Susanna Júnior R were responsible for the manuscript revision and orientation.

## Figures and Tables

**Table 1:** Distribution of Visual Acuity Without Correction*, n (%).

	Right Eye	Left Eye	Total
1.00	363 (3336)	368 (34.1)	731 (33.8)
0.90	173 (16.0)	171 (15.8)	344 (15.9)
0.80	76 (7.0)	83 (7.7)	159 (7.3)
0.70	96 (8.9)	90 (8.3)	186 (8.6)
0.60	82 (7.6)	72 (6.7)	154 (7.1)
0.50	85 (7.9)	77 (7.1)	162 (7.5)
0.40	54 (5.0)	62 (5.7)	116 (5.3)
0.30	49 (4.5)	43 (4.0)	92 (4.2)
0.20	38 (3.5)	41 (3.8)	79 (3.6)
0.15	17 (1.6)	16 (1.5)	33 (1.5)
0.10	14 (1.4)	19 (1.8)	33 (1.5)
Finger count	3 (0.3)	8 (0.7)	11 (0.5)
Hand movement	0	2 (0.2)	2 (0.1)
Non-cooperation or inability to evaluate	30 (2,8)	28 (2.6)	58 (2.6)
Total	1080 (100)	1080 (100)	2160 (100)

* Values on the decimal score on the Snellen Visual Acquity (VA) Chart.

**Table 2:** Distribution of Ocular Movement Disorders.

	N (%)
X(T) - Exoforia	16 (13.6)
E(T) - Esoforia	7 (5.9)
X - Intermitent exotropia	22 (18.8)
E - Intermitent esotropia	8 (6.8)
ET - Manifest esotropia	32 (27.3)
XT - Manifest exotropia	32 (27.3)
Total	117 (100)

**Table 3:** Diagnoses and Alterations detected.

	n° of occurrences
Strabismus	117
Ambliopia	54
Myopic fundus	8
RPE rarefaction	5
Optic disc pallor	4
Increased disc/escavation relation	2
Crowded disc	2
Persistent hyperplastic primary vitreous	1
Persistent myelinated fibers in the retina	1
Choriorretinitis scar	1
Tilted disc	1
Optic disc dysplasia	1
Fundus albinus	1
Peripapillary atrophy	1
Retinal detachment	1
Total number of fundoscopy alterations	29
Conjunctival papillae	8
Microcornea	3
Corneal opacifications	3
Conjunctival melanocitoses	2
Iris transillumination	1
Persistent pupillary membrane	1
Distichiasis	1
Crystalline vacuoles	1
Total number of biomicroscopic alterations	20
Total	220
